# A Local Oscillator Phase Compensation Technique for Ultra-Wideband Stepped-Frequency Continuous Wave Radar Based on a Low-Cost Software-Defined Radio †

**DOI:** 10.3390/s21030780

**Published:** 2021-01-24

**Authors:** Kazunori Takahashi, Takashi Miwa

**Affiliations:** 1 OYO Corporation, Miyahara-cho 1-66-2, Kita-ku, Saitama 331-0812, Japan; 2 Faculty of Science and Technology, Gunma University, Tenjin-cho 1-5-1, Kiryu, Gunma 376-8515, Japan; miwa@gunma-u.ac.jp

**Keywords:** local oscillator (LO), phase compensation, radar, software-defined radio (SDR), stepped-frequency continuous wave (SFCW), ultra-wideband (UWB)

## Abstract

The paper discusses a way to configure a stepped-frequency continuous wave (SFCW) radar using a low-cost software-defined radio (SDR). The most of high-end SDRs offer multiple transmitter (TX) and receiver (RX) channels, one of which can be used as the reference channel for compensating the initial phases of TX and RX local oscillator (LO) signals. It is same as how commercial vector network analyzers (VNAs) compensate for the LO initial phase. These SDRs can thus acquire phase-coherent in-phase and quadrature (I/Q) data without additional components and an SFCW radar can be easily configured. On the other hand, low-cost SDRs typically have only one transmitter and receiver. Therefore, the LO initial phase has to be compensated and the phases of the received I/Q signals have to be retrieved, preferably without employing an additional receiver and components to retain the system low-cost and simple. The present paper illustrates that the difference between the phases of TX and RX LO signals varies when the LO frequency is changed because of the timing of the commencement of the mixing. The paper then proposes a technique to compensate for the LO initial phases using the internal RF loopback of the transceiver chip and to reconstruct a pulse, which requires two streaming: one for the device under test (DUT) channel and the other for the internal RF loopback channel. The effect of the LO initial phase and the proposed method for the compensation are demonstrated by experiments at a single frequency and sweeping frequency, respectively. The results show that the proposed method can compensate for the LO initial phases and ultra-wideband (UWB) pulses can be reconstructed correctly from the data sampled by a low-cost SDR.

## 1. Introduction

Radar in the microwave frequency range is finding an increasing number of applications in many fields, such as imaging buried and concealed objects non-destructively, automotive, biomedical, and material characterization. The measurement configuration and set-up may need to be customized and optimized for a specific application to maximize the performance before going into production. Therefore, prototyping a radar system is an important process for exploring new applications. A flexible instrument is required for prototyping because various measurement parameters need to be tuned. Stepped-frequency continuous wave (SFCW) radar based on a commercial vector network analyzer (VNA) has been commonly employed for this purpose, especially for ultra-wideband (UWB) and near-range radar applications, as it allows users to easily control various parameters and set-up such as the frequency range and measurement sequences. However, commercial VNAs usually have too many functions, which are not always necessary to configure a simple SFCW radar, and are therefore often too expensive for only prototyping; moreover, their typical large size and heavy weight may constrain the system configuration. For these reasons, although commercial VNAs are suitable for prototyping radar systems, they may not always be the best choice. Therefore, other cheaper, smaller, and more lightweight solutions would be appreciated.

Software-defined radio (SDR) is a tool that is primarily intended to be used to test and prototype various communication applications such as wireless communications and the Global Navigation Satellite System (GNSS). For example, these include mobile telecommunication [1,2], multi-static multiple-input and multiple-output (MIMO) communication [3,4,5], Wi-Fi [6], characterizing radio-frequency identification (RFID) tags [7,8], prototyping a global positioning systems (GPS) receiver [9,10], locating satellites [11], and indoor positioning [12]. The use of SDR is becoming popular because of the fully digital in-phase and quadrature (I/Q) interface, which provides flexibility in signal generation and postprocessing of received signals. An SDR typically employs a direct conversion (homodyne, zero-IF) transmitter and receiver architecture to make it simple, compact, and inexpensive compared to general-purpose RF instruments such as the VNA and the spectrum analyzer. For example, the price of low-cost SDRs can be one-hundredth that of mid-grade VNAs. Therefore, it is suitable for prototyping.

Some previous studies have prototyped radars using SDRs for various applications. For example, a frequency-modulated continuous wave (FMCW) radar for exploring polar ice sheet [13], a random noise radar for through-the-wall surveillance [14], multi-static MIMO radar for monitoring moving targets [15], a waveform adaptive MIMO radar [16], and a forward scatterer radar for profile reconstruction [17]. It is straightforward to build an FMCW radar using the instantaneous (modulation) bandwidth of an SDR at a selected carrier or local oscillator (LO) frequency (see, e.g., in [13,15,18]) and an SFCW or orthogonal frequency division multiplexing (OFDM) radar within the instantaneous bandwidth (see, e.g., in [19]). Radars using the instantaneous bandwidths of SDRs require only one TX and RX streaming to obtain a waveform. The TX and RX streaming must be synchronized, but it can be easily done by using a function provided by the API of SDRs, e.g., by using timestamps. Therefore, it is simple to build an UWB radar using an SDR if the SDR offers a wide enough instantaneous bandwidth. For example, the authors of [20,21] constructed an SDR with 800 MHz bandwidth, which can achieve approximately 20 cm resolution. An SDR used for a random noise radar system in [14] has 400 MHz instantaneous bandwidth and achieved 37.5 cm range resolution. These wideband SDRs are typically more expensive than low-cost ones in the market that this work is targeting. Low-cost SDRs are intended to be used primarily for communication, the instantaneous bandwidth is thus typically not very wide. The pulse width cannot be narrow enough to achieve a high time or range resolution by using the instantaneous bandwidth of low-cost SDRs. For example, SDR-based radars using 25 MHz modulation frequency bandwidth achieved a pulse width of approximately 10 m [18,19]. The pulse width obtained in this manner is not narrow enough for a near-range radar.

By contrast, SFCW can use the full carrier (modulation) frequency bandwidth of SDR, which is usually wide to adapt to a wide range of frequency bands for various applications. With SFCW, it is thus possible to construct a UWB radar, and it is more suitable for near-range radar. The key operation of the SFCW radar is to coherently measure the amplitude and phase of CW signals at a number of frequencies. However, the phase of the CW signals cannot be directly obtained due to the unknown initial phase the of LO signals when low-cost SDRs, which have only one TX and RX channel, are used. There are a few examples of SFCW radar based on SDRs with multiple RX channels [22,23]. Such SDRs usually cost significantly more than low-cost ones. Employing RF switches is another way to retrieve the phase coherently [24,25]; however, it also costs for the additional components. The paper proposes an alternative method of circumventing the incoherent phase by using the internal RF loopback of the transceiver chip and with no additional components unlike previous studies, thus while retaining the important feature of SDRs—low cost—is retained. Additionally, the cause and mechanism of the random phase in relation with the proposed method is discussed thoroughly.

The next section discusses the mechanism underlying the unknown initial phases of the LO signals, which are contained in sampled CW signals and proposes a compensation technique. Section 3 presents experiment results that illustrate the effect of the unknown initial phase. In addition, an experiment with a model of SFCW radar using the cable through configuration is presented to demonstrate how the proposed method works and how a short pulse can be obtained. Note that the work presented here was developed using a commercial off-the-shelf and low-cost SDR, Nuand bladeRF, which employs the RF transceiver LimeMicro LMS6002D. The method of implementing the proposed method may depend upon the model of SDR used. Nuand bladeRF is chosen in this work because it has I/Q input/output, full duplex capability, and a relatively wider carrier frequency bandwidth compared with other low-cost SDRs available in the market. These are necessary features to build a near-range UWB SFCW radar. Note also that the paper extends the previous work [26] and also provides more comprehensive explanations of the unknown initial phase of LO signals and experiment results.

## 2. SDR-Based SFCW Radar

The working principle of SFCW radar is reviewed and how it can be implemented into an SDR is discussed in this section to illustrate the issue of the LO initial phase. To address the issue, a solution using the internal RF loopback channel of the transceiver chip is presented.

### 2.1. Working Principle

The SDR consists of a transmitter (TX) path, which modulates baseband signals by a local oscillator (LO) and up-converts them to an RF frequency, and a receiver (RX) path, which demodulates received signals by another LO and down-converts them to a baseband signal, i.e., a direct conversion (also known as homodyne and zero-IF) detector is used, in contrast to the super-heterodyne detector used in commercial VNAs. Because DC offset and I/Q imbalance can cause a serious deterioration in performance in the direct conversion detector, an I/Q baseband signal $x_{b}\left(t\right)$ at a frequency $\omega_{b}$, which corresponds to an intermediate frequency (IF) signal, is digitally generated in the host computer as

(1) $$\begin{array}{c} x_{b}\left(t\right)=\exp\left(j\omega_{b}t\right) \end{array}$$

The generated digital baseband signal $x_{b}\left(t\right)$ is then transferred to the SDR. Note that for simplicity the amplitude of the I/Q baseband signal is assumed to be 1 here; however, one may want to scale the signal so that it fully spans the dynamic range of digital-to-analogue converter (DAC) of the SDR to have a better signal-to-noise ratio. The transferred I/Q baseband signal is converted to an analogue signal for the in-phase and quadrature channels separately at the DACs in the SDR. The signal is then mixed by an image rejection mixer (IRM) or I/Q mixer with the LO signal for the in-phase channel and with the 90${}^{\circ }$ phase-shifted LO signal for the quadrature channel at the carrier frequency $\omega_{\mathrm{LO}}$ which is equivalent to multiplying with $x_{\mathrm{LO}}\left(t\right)=\exp\left(j\omega_{\mathrm{LO}}t\right)$, as

(2) $$\begin{array}{cc} x(t) & =x_{b}\left(t\right)\cdot x_{\mathrm{LO}}\left(t\right)\\ \; & =\exp\left(j\omega_{b}t\right)\cdot \exp\left(j\omega_{\mathrm{LO}}t\right)\\ \; & =\exp\{j\left(\omega_{\mathrm{LO}}+\omega_{b}\right)t\} \end{array}$$

The baseband signal is up-converted to the frequency $\omega_{\mathrm{LO}}+\omega_{b}$ by the mixing as seen in the above equation. The mixed signal at the frequency $\omega_{\mathrm{LO}}+\omega_{b}$ is then transmitted to the TX antenna from the TX port and radiated. The RX signal y(t) received by the RX antenna and entering the RX port can be modeled as the TX signal with changes in amplitude $A_{\omega }$ and phase $\phi_{\omega }$ due to the signal propagation and reflection at the frequency $\omega =\omega_{\mathrm{LO}}+\omega_{b}$:

(3) $$\begin{array}{cc} y(t) & =A_{\omega }x\left(t\right)\exp\left(j\phi_{\omega }\right)\\ \; & =A_{\omega }\exp\left\{j\left(\omega_{\mathrm{LO}}+\omega_{b}\right)t+j\phi_{\omega }\right\} \end{array}$$

The received signal y(t) is mixed with the complex conjugate of the RX LO signal $y_{\mathrm{LO}}\left(t\right)=\exp\left(j\omega_{\mathrm{LO}}t\right)$ at the carrier frequency $\omega_{\mathrm{LO}}$:

(4) $$\begin{array}{cc} y_{b}\left(t\right) & =y\left(t\right)\cdot y_{\mathrm{LO}}^{*}\left(t\right)\\ \; & =A_{\omega }\exp\left\{j\left(\omega_{\mathrm{LO}}+\omega_{b}\right)t+j\phi_{\omega }\right\}\exp\left(-j\omega_{\mathrm{LO}}t\right)\\ \; & =A_{\omega }\exp\left(j\omega_{b}t+j\phi_{\omega }\right) \end{array}$$

where ${}^{*}$ denotes the complex conjugate. The received signal is down-converted to the RX baseband signal at the baseband (IF) frequency $\omega_{b}$. The RX baseband signal is sampled by analogue-to-digital converters (ADCs) separately for the in-phase and quadrature channels, and is digitally output from the SDR to the host computer. The baseband signal can then be digitally filtered and mixed with a CW signal with frequency $\omega_{b}$, i.e., $\exp(-j\omega_{b}t)$, to further down-convert to DC, which corresponds to the complex amplitude, i.e., amplitude and phase, or I/Q data at the frequency $\omega =\omega_{\mathrm{LO}}+\omega_{b}$. However, due to noise, LO leakage, I/Q imbalance or various reasons, the mixed signal may not be down-converted exactly to DC and may fluctuate. Therefore, the sample mean of the signal is calculated to obtain stable I/Q data:

(5) $$\begin{array}{cc} S(\omega ) & =\frac{1}{T}\int_{T}y_{b}\left(t\right)\exp\left(-j\omega_{b}t\right)\;dt\\ \; & =\frac{1}{T}\int_{T}A_{\omega }\exp\left(j\phi_{\omega }\right)\;dt\\ \; & =A_{\omega }\exp\left(j\phi_{\omega }\right) \end{array}$$

Repeating the operation for various carrier (LO) frequencies $\omega_{\mathrm{LO}}$ allows us to collect the complex amplitudes for a wide range of TX frequencies $\omega =\omega_{\mathrm{LO}}+\omega_{b}$, which correspond to the forward transmission *S*-parameter, i.e., $S_{21}$, between the TX and RX ports. Therefore, the impulse response can be obtained by the inverse Fourier transform of the obtained complex amplitudes for a range of frequencies. This is how SFCW radar acquires a UWB pulse.

### 2.2. Implementation in SDR and Compensation of the Initial Phase of the LO Signal

**Figure 1:**
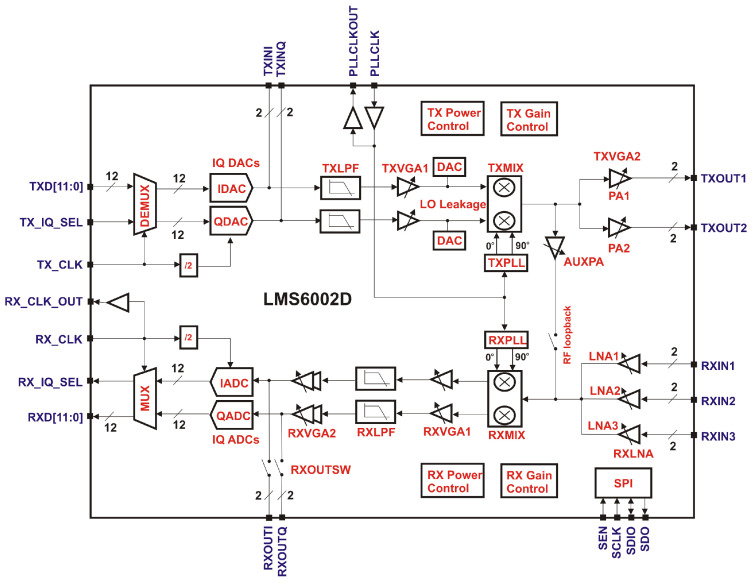
Functional block diagram of an RF transceiver, LimeMicro LMS6002D, which is employed by a low-cost SDR, Nuand bladeRF [28]. bladeRF supplies 38.4 MHz reference clock to the chip.

**Figure 2:**
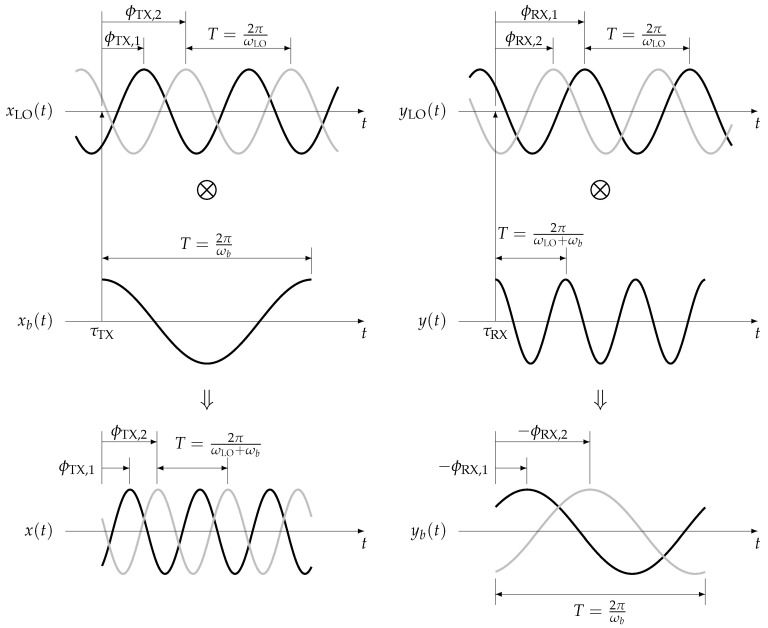
Schematic illustration of phase difference in TX signal and RX baseband signals due to the initial phases of LO signals. A TX baseband signal $x_{b}\left(t\right)$ (middle left) is mixed at time $\tau_{\mathrm{TX}}$ with a TX LO signal $x_{\mathrm{LO}}\left(t\right)$ (top left). Depending on the timing of the mixing, the TX LO signal exhibits the initial phase $\phi_{\mathrm{TX},1}$ (signal in black) or $\phi_{\mathrm{RX},2}$ (signal in gray). The mixed signals x(t) (bottom left) exhibit different initial phases, which correspond to the phases of the TX LO signals relative to that of the TX baseband signal. The same applies to signals in the RX path (signals on the right side); a RX signal y(t) (middle right), which has zero initial phase in this example, begins to be received at time $\tau_{\mathrm{RX}}$ and commences to be mixed with a RX LO signal $y_{\mathrm{LO}}\left(t\right)$ (top right). The two RX LO signals with different initial phases $\phi_{\mathrm{RX},1}$ (signal in black) and $\phi_{\mathrm{RX},2}$ (signal in gray) yield the RX baseband signals $y_{b}\left(t\right)$ (bottom right) with different phases of which the difference corresponds to the phase difference of the RX LO signals. Note that only the in-phase components of signals are drawn for simplicity. Furthermore, the LO frequency may be much higher than baseband frequency, i.e., $\omega_{\mathrm{LO}}\gg \omega_{b}$, e.g., three to five orders, in reality, but they are illustrated to be comparable for clarity.

The working principle of SFCW radar discussed in the previous section is implemented into a low-cost SDR, Nuand bladeRF. The specification is summarized in Table 1. The SDR uses an RF transceiver, LimoMicro LMS6002D, which has two phase-locked loops (PLLs) as separate LOs for the TX and RX as shown in Figure 1. The outputs of the PLLs, i.e., carrier or LO signals, are mixed with the TX baseband signal $x_{b}\left(t\right)$ in the TX path and with the RX signal y(t) in the RX path. The phase of the mixed signals contains the initial phases of the LO signals $\phi_{\mathrm{TX}}$ and $\phi_{\mathrm{RX}}$, which vary every time depending on the timing of the mixing. The situation is schematically illustrated in Figure 2. A TX baseband signal $x_{b}\left(t\right)$ (middle left) is entering the mixer at time $\tau_{\mathrm{TX}}$ and is mixed with a TX LO signal $x_{\mathrm{LO}}\left(t\right)$ (top left). Because the TX LO signal is free-running, the phase at the time $\tau_{\mathrm{TX}}$ cannot be controlled, and thus the TX LO signal appears to have an initial phase that depends entirely on the phase at the commencement of mixing $\tau_{\mathrm{TX}}$. In the figure, the TX LO signal could be in black or gray depending on the timing, for example. The initial phase can be represented by that relative to the time when the mixing begins, $\tau_{\mathrm{TX}}$; i.e., $\phi_{\mathrm{TX}}=\phi_{\mathrm{TX},1}$ for the signal in black or $\phi_{\mathrm{TX},2}$ for the signal in gray. The TX LO signal can thus be written explicitly with the initial phase $\phi_{\mathrm{TX}}$ as

(6) $$\begin{array}{c} x_{\mathrm{LO}}\left(t\right)=\exp\left(j\omega_{\mathrm{LO}}t+j\phi_{\mathrm{TX}}\right) \end{array}$$

Mixing with the TX LO signal $x_{\mathrm{LO}}\left(t\right)$ and TX baseband signal $x_{b}\left(t\right)$ results in the TX signal x(t) with the initial phase of the TX LO signal $\phi_{\mathrm{TX}}$:

(7) $$\begin{array}{cc} x(t) & =\exp\left(j\omega t\right)\cdot \exp\left(j\omega_{\mathrm{LO}}t+j\phi_{\mathrm{TX}}\right)\\ \; & =\exp\{j\left(\omega_{\mathrm{LO}}+\omega_{b}\right)t+j\phi_{\mathrm{TX}}\} \end{array}$$

As one can see in the figure, the TX signal x(t) (bottom left) retains the initial phase of the TX LO signal $\phi_{\mathrm{TX}}$. The TX signals therefore exhibit different phases for ones produced by the TX LO signals with the initial phases $\phi_{\mathrm{TX},1}$ and $\phi_{\mathrm{TX},2}$ as shown in black and gray in the figure. As seen in the above equation and illustrated in the figure, the initial phases of the mixed TX signals x(t) relative to the commencement of mixing correspond to those of TX LO signals, $\phi_{\mathrm{TX},1}$ and $\phi_{\mathrm{TX},2}$ in the figure, assuming the initial phase of the TX baseband signal $x_{b}\left(t\right)$ is zero.

The same applies to the RX path: the RX signal y(t) (middle right) is mixed with an RX LO signal $y_{\mathrm{LO}}\left(t\right)$ (top right) starting at time $\tau_{\mathrm{RX}}$. The RX LO signal has an initial phase depending on the timing, e.g., $\phi_{\mathrm{RX},1}$ or $\phi_{\mathrm{RX},2}$ in the figure. The RX LO signal $y_{\mathrm{LO}}\left(t\right)$ with the initial phase $\phi_{\mathrm{RX}}$ can thus be written as

(8) $$\begin{array}{c} y_{\mathrm{LO}}\left(t\right)=\exp\left(j\omega_{\mathrm{LO}}t+j\phi_{\mathrm{RX}}\right) \end{array}$$

The mixing of the RX signal y(t) with the RX LO signal $y_{\mathrm{LO}}\left(t\right)$ results in the RX baseband signal $y_{b}\left(t\right)$ (bottom right) down-converted to the frequency $\omega_{b}$. The mixed RX signal again retains the initial phase of the RX LO signal as shown in black and gray in the figure. Note that the initial phase of the RX baseband signal corresponds to the additive inverse of the that of the RX LO signal because the complex conjugate of the RX LO signal is mixed to down-convert. Because the RX signal can be modeled as the amplitude-altered and phase-shifted version of the TX signal x(t), respectively, by $A_{\omega }$ and $\phi_{\omega }$ as shown in Equation (3), the RX baseband signal $y_{b}\left(t\right)$ also carries the initial phase of the TX LO signal $\phi_{\mathrm{TX}}$ in addition to that of the RX LO signal $\phi_{\mathrm{RX}}$:

(9) $$\begin{array}{cc} y_{b}\left(t\right) & =y\left(t\right)\exp\left(-j\omega_{\mathrm{LO}}t-j\phi_{\mathrm{RX}}\right)\\ \; & =A_{\omega }\left(t\right)\exp\left(j\phi_{\omega }\right)\exp\left(-j\omega_{\mathrm{LO}}t-j\phi_{\mathrm{RX}}\right)\\ \; & =A_{\omega }\exp\left(j\omega_{b}t+j\phi_{\omega }+j\phi_{\mathrm{TX}}-j\phi_{\mathrm{RX}}\right) \end{array}$$

As mentioned, the TX and RX LO signals $x_{\mathrm{LO}}\left(t\right)$ and $y_{\mathrm{LO}}\left(t\right)$ are freely running, and the initial phases of the LO signals $\phi_{\mathrm{TX}}$ and $\phi_{\mathrm{RX}}$ depend upon the timing of the commencement of the mixing. The initial phases of the TX signal x(t) and RX baseband signal $y_{b}\left(t\right)$ would become zero and the I/Q data obtained by simply applying the processing discussed in the previous section become phase coherent if the TX and RX mixing begins where the initial phases of LO signals were zero. However, it may not be viable on most of low-cost SDRs as it requires the very fine timing control. For example, the LO signals could be in gigahertz frequency range whereas the TX baseband signal may be sampled in megahertz sampling frequency. The sample shift of the TX baseband signal is not fine enough to control the initial phase of the TX LO signal. The timing of RX signals coming into the RX port cannot be controlled in general as they are not predictable in radar application. Therefore, the terms of the LO initial phases in Equation (9) are unknown and vary every time; however, they need to be either known or canceled to retrieve the phase of the complex amplitude $\phi_{\omega }$.

The most straightforward way to compensate for the initial phases of the LO signals is to employ an additional receiver, commonly referred to as a reference receiver, which is implemented in commercial VNAs [29] and SDRs that have two or more synchronized receivers. Reference receiver is connected at the output of an LO source to directly sample each LO signal. However, only one receiver per port is available in the case of low-cost SDRs, which makes it difficult for such SDRs to be used for SFCW radar. A few previous studies attempted to circumvent the problem by employing additional components. For example, the authors of [24] used two single pole double throw (SPDT) switches, one each at the TX and RX ports, to configure two signal paths, which are a device under test (DUT) path with antennas and a reference path with cable through. The authors of [22,23] employed an SDR with two RX ports, and TX signals can thus be directly input to the second RX port by using a splitter at the TX port. The authors of [25] performed measurements in reflection mode (i.e., $S_{11}$) by employing a circulator, SPDT, and 50 Ω terminator. These previous works succeeded to obtain phase-coherent I/Q data and UWB pulses. However, employing additional components increases the overall cost and complexity of a system which may not be taking the full advantage of using an SDR and may be better to avoid if possible.

In this study, the internal RF loopback shown in the functional diagram of the transceiver chip in Figure 1 is used. In this way, no additional components are needed. Instead, TX and RX streaming need to be performed twice to measure two paths, i.e., the DUT and the internal RF loopback paths. The RX baseband signal through the loopback path has the same initial phases as that in the DUT path (a detailed explanation is provided later), but a different delay $\phi_{\omega ,\mathrm{LB}}$ due to the difference of the path lengths:

(10) $$\begin{array}{c} y_{b,\mathrm{LB}}\left(t\right)=A_{\omega ,\mathrm{LB}}\exp\left(j\omega_{b}t+j\phi_{\omega ,\mathrm{LB}}+j\phi_{\mathrm{TX}}-j\phi_{\mathrm{RX}}\right) \end{array}$$

The unknown phase can be canceled by diving the RX baseband signal in the DUT path $y_{b}\left(t\right)$ by the loopback path $y_{b,\mathrm{LB}}\left(t\right)$ as follows to obtain the phase coherent RX baseband signal ${\bar{y}}_{b}\left(t\right)$:

(11) $$\begin{array}{cc} {\bar{y}}_{b}\left(t\right) & =\frac{y_{b}\left(t\right)}{y_{b,\mathrm{LB}}\left(t\right)}\\ \; & =\frac{A_{\omega }}{A_{\omega ,\mathrm{LB}}}\exp\left(j\phi_{\omega }-j\phi_{\omega ,\mathrm{LB}}\right) \end{array}$$

The frequency component at the baseband frequency $\omega_{b}$ can also be cancelled and down-converted to the DC. The complex amplitude at $\omega =\omega_{\mathrm{LO}}+\omega_{b}$ can therefore be obtained by taking the average:

(12) $$\begin{array}{cc} S(\omega ) & =\frac{1}{T}\int_{T}{\bar{y}}_{b}\left(t\right)\;dt\\ \; & =\frac{A_{\omega }}{A_{\omega ,\mathrm{LB}}}\exp\left(j\phi_{\omega }-j\phi_{\omega ,\mathrm{LB}}\right) \end{array}$$

The obtained amplitude $\frac{A_{\omega }}{A_{\omega ,\mathrm{LB}}}$ and phase $\phi_{\omega }-\phi_{\omega ,\mathrm{LB}}$ are both relative to those of the RF loopback path, $A_{\omega ,\mathrm{LB}}$ and $\phi_{\omega ,\mathrm{LB}}$. Similar to the response calibration in the VNA, the amplitude and phase of a DUT alone, $A_{\omega }$ and $\phi_{\omega }$, can be obtained by dividing the data using a cable through data, which further cancels the frequency response of the cables and also the loopback path, $A_{\omega ,\mathrm{LB}}$ and $\phi_{\omega ,\mathrm{LB}}$.

The previous work [26] digitally down-converts the sampled baseband signals in DUT and loopback channels separately by mixing a CW signal in the host computer, and the down-converted signal in the DUT channel is divided by that in loopback channel. The way thus performs the down-conversion and LO phase compensation separately. The method presented in this paper combines the steps by dividing DUT baseband signal by loopback channel. They are mathematically identical.

As mentioned earlier, the initial phases of the TX and RX LO signals must change every time. However, the unknown phase term in Equation (9), i.e., $\phi_{\mathrm{TX}}-\phi_{\mathrm{RX}}$, is the relative phase difference of the TX and RX LO signals, each initial phase $\phi_{\mathrm{TX}}$ and $\phi_{\mathrm{RX}}$ can be defined relative to the input timings of the TX baseband signal $x_{b}\left(t\right)$ and RX signal y(t), respectively, and the timing relative to another must remain the same if the timing relationship between the TX and RX LO signals is kept unchanged. Therefore, the absolute phases of the LO signals at the $\tau_{\mathrm{TX}}$ and $\tau_{\mathrm{RX}}$ may change every time, but their difference $\phi_{\mathrm{TX}}-\phi_{\mathrm{RX}}$ remains the same, and the division by RF loopback data thus works. It requires the TX and RX LO signals to be synchronized. In the case of LMS6002D, the two PLLs for the TX and RX share the same clock signal. The difference of the initial phases between TX and RX LO signals changes if the frequencies of the TX and RX LO signals are re-tuned because it changes the relative phase between the LOs. Therefore, the two TX and RX streaming for the DUT and loopback paths must be performed one right after another without frequency tuning. The method does not work if the TX and RX LO signals are set to different frequencies because the relative phase between the signals with different frequencies constantly varies.

## 3. Experiments

The method of compensating for the initial phases of LO signals by using the internal RF loopback of a transceiver chip as the reference channel discussed in the previous section is demonstrated by experiments using Nuand bladeRF x40.

### 3.1. At a Single Frequency

The relationship between the TX and RX LO signal phases discussed in the previous section is first demonstrated at a single frequency in this section.

**Figure 3:**
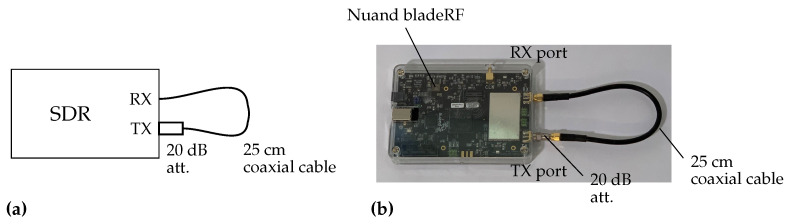
Measurement set-up of the experiment at a single frequency with 25 cm coaxial cable and 20 dB attenuator. (**a**) Schematic illustration and (**b**) photo.

The TX and RX ports of bladeRF are connected by a 25 cm cable with a 20 dB attenuator as shown in Figure 3. A TX baseband signal at 1 MHz is generated in the host computer and input to the SDR. The TX and RX LO frequencies are both set to 1 GHz. The set-up is summarized in Table 2. TX and RX streaming, which are synchronized based on the FPGA timestamp, are repeated 10 times under two conditions: one with the LO frequency tuned to 1 GHz before every streaming and the other with the LO frequency tuned only once before the first streaming.

**Figure 4:**
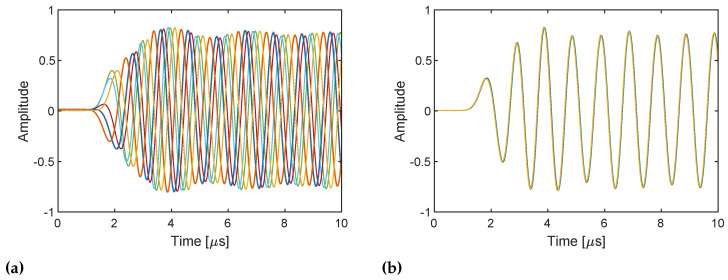
In-phase component of the RX baseband signals at 1 MHz for 10 repetitions of TX and RX streaming (**a**) with frequency tuning every time and (**b**) with frequency tuning only once at the beginning. TX and RX LO frequencies are both set to 1 GHz. The baseband signals exhibit different phase when frequency is re-tuned.

**Figure 5:**
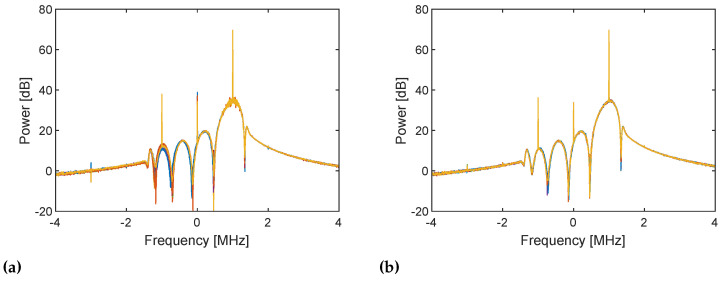
Power spectra of the RX baseband signals for 10 repetitions of TX and RX streaming (**a**) with frequency tuning every time and (**b**) with frequency tuning only once at the beginning. Both cases give the same power spectra for 10 repetitive streams because the envelope of the baseband signals are the same although the phases are different (Figure 4). Frequency components at 1 MHz are the baseband signals and are the desired components. Components at DC are caused by DC offset, and those at −1 MHz are the image of baseband signals caused by I/Q imbalance.

**Figure 6:**
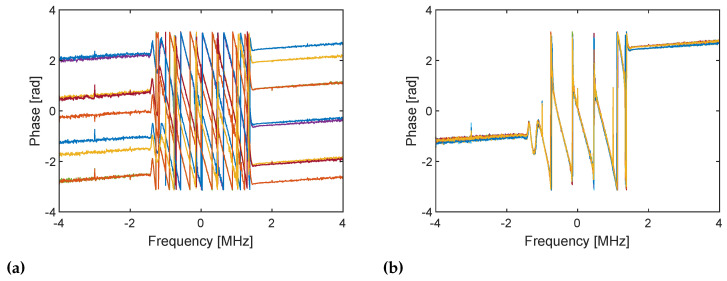
Phase spectra of the RX baseband signals for 10 repetitions of TX and RX streaming (**a**) with frequency tuning every time and (**b**) with frequency tuning only once at the beginning. Subfigure (**a**) shows randomly varying phases at 1 MHz whereas phases at 1 MHz are consistent in subfigure (**b**).

The in-phase component of the received RX baseband samples for 10 repetitions is shown in Figure 4. All the baseband signals consistently start being received at approximately 1.5 μs in both cases. This indicates that the TX and RX streaming are synchronized. This yields the identical power spectra shown in Figure 5. However, the phase of the received signals varies when frequency tuning is performed every time (Figure 4a), and the signals thus exhibit randomly varying phase spectra (Figure 6a). This may be caused by the slightly different timings of the frequency lock of the TX and RX PLLs, which changes the relative phase between the TX and RX LO signals. By contrast, when frequency tuning is performed only once at the beginning (Figure 4b), a consistent phase is obtained, which yields the identical phase spectra shown in Figure 6b. The phase of the TX and RX LO signals where the signals start being mixed (i.e., $\phi_{\mathrm{TX}}$ and $\phi_{\mathrm{RX}}$ in Figure 2) must be different for the 10 repetitions of streaming. However, because frequency tuning is not performed during the repetition, the relative phase between the TX and RX LO signals is kept unchanged. Therefore, the unknown phase term $\phi_{\mathrm{TX}}-\phi_{\mathrm{RX}}$ in Equation (9) is also kept unchanged, and all the RX baseband signals have the same phase. The result verifies the discussion in the previous section and implies that phase compensation using a loopback signal works as long as the two streaming for the DUT and internal RF loopback paths are performed immediately in quick succession without frequency tuning.

### 3.2. Frequency Sweeping

To construct a UWB radar in the step frequency manner, frequency responses in a wide frequency range have to be collected. The LO frequency of the SDR must thus be swept through a wide frequency range as SDRs typically have a narrow instantaneous bandwidth that is not enough to reconstruct a short pulse. This section demonstrates sweeping the LO frequency in the cable through configuration and shows that an SDR can be used to build a UWB radar.

**Figure 7:**
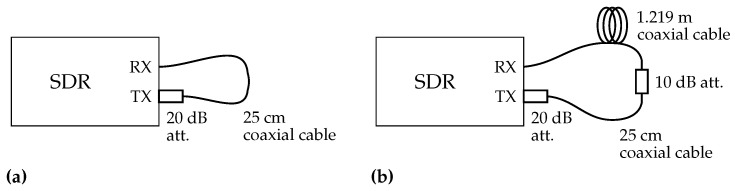
Measurement set-up of the frequency sweeping experiments. (**a**) Cable through configuration with 25 cm coaxial cable and 20 dB attenuator, which is the same as the previous experiment shown in Figure 3, and (**b**) with additional 1.219 m (4 ft) coaxial cable and 10 dB attenuator.

The TX baseband signal fixed at 1 MHz is again generated in the host computer and transferred to the SDR. The TX and RX LO frequencies are swept so that the frequencies of the signals transmitted from the TX port (i.e., the sum of the baseband and LO frequencies) range from 250 MHz to 3.75 GHz, which is almost the full bandwidth of the LO frequency of the SDR, with a step size of 50 MHz. At each LO frequency, two streaming for the DUT and loopback paths are performed immediately in quick succession without frequency tuning, as discussed in the previous section. The TX and RX sampling frequencies and the number of baseband samples are respectively set to 32 MHz and 4096. In this experiment, the DUT is a 25 cm coaxial cable and 20 dB attenuator that configure cable through between the TX and RX ports as shown in Figure 7a and as in the test described in the previous section. Moreover, another measurement is performed with an additional 1.219 m (4 ft) cable and 10 dB attenuator extending the cable through set-up as shown in Figure 7b. The set-up of the experiment is summarized in Table 2.

**Figure 8:**
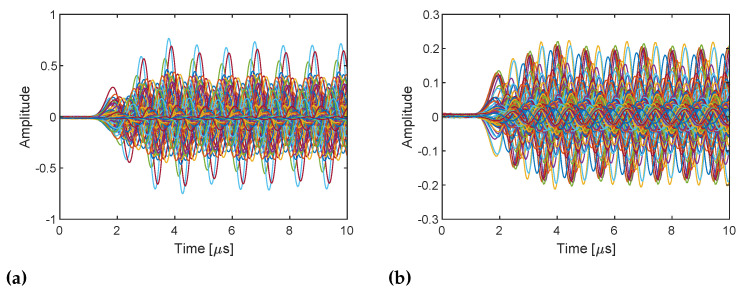
In-phase components of the RX baseband signals for 71 TX and RX LO frequencies in (**a**) the DUT channel (cable through with 25 cm cable and 20 dB attenuator) and (**b**) the reference channel (internal RF loopback). All baseband signals start being received at approximately 1.5 μs which indicates that the TX and RX streaming are synchronized.

**Figure 9:**
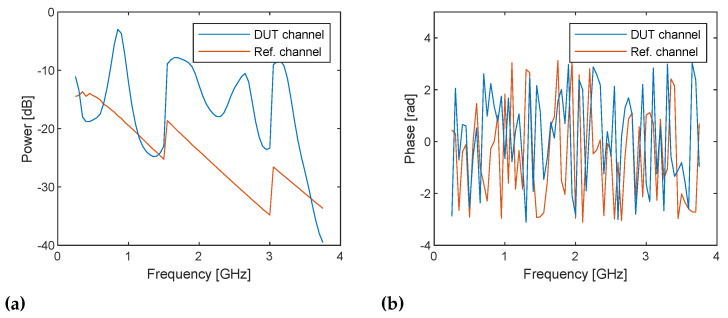
(**a**) Power and (**b**) phase spectra of the IQ data obtained directly from the sampled RX baseband signals measured at frequencies from 250 MHz to 3.75 GHz with a step size of 50 MHz in the DUT channel with 25 cm cable and 20 dB attenuator. Note that the discontinuities at 1.5 and 3 GHz in the power spectra are caused by gain adjustment. The power spectra (**a**) shows the frequency response of the DUT and loopback channels whereas phase spectra (**b**) look random because they contain the initial phase of LO signal, which varies randomly.

**Figure 10:**
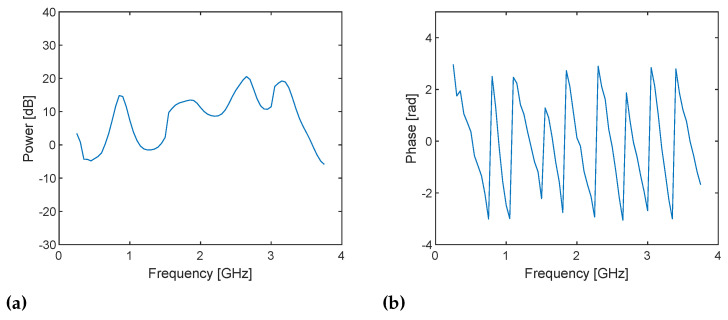
(**a**) Power and (**b**) phase spectra of the I/Q data obtained by dividing the baseband signals in the DUT channel by those in the RF loopback channel and taking the average at frequencies from 250 MHz to 3.75 GHz with a step size of 50 MHz for the cable through with 25 cm cable and 20 dB attenuator. Randomly varying phase in Figure 9b become linear. Note that the discontinuities at 1.5 and 3 GHz in the power spectrum are caused by gain adjustment.

Figure 8 shows the in-phase components of RX baseband signals sampled with the 71 LO frequencies in the DUT and reference channels in the configuration with a 25 cm cable and 20 dB attenuator (Figure 7a). The baseband signals start being received at approximately 1.5 μs in both channels. It can also be observed that baseband signals have a DC offset at some frequencies. The sampled signals are bandpass filtered at the baseband frequency (i.e., $\omega_{b}=$ 1 MHz) digitally in the host computer to suppress the influence of the image component and DC offset due to the I/Q imbalance of the system, which can be observed in the previous experiment (Figure 5), and to remove noise. I/Q data are obtained by digitally mixing the filtered baseband signals and 1 MHz continuous wave and taking the average from 1/4 to 3/4 of the full-time range (i.e., 1025–3072 samples), which is the steady part. Figure 9 shows the power and phase spectra of the I/Q data with respect to the TX frequency $\omega =\omega_{\mathrm{LO}}+\omega_{b}$. The power spectra show the frequency responses of the DUT and RF loopback channels, which seem reasonable. Note that the discontinuities at 1.5 and 3 GHz are caused by changing the gains. On the other hand, the phase spectra show random variations because the sampled baseband signals are not phase coherent and contain the initial phases of TX and RX LO signals, which randomly change at each streaming. As shown in Equation (11), the initial phases of LO signals are canceled by dividing the samples of the RX complex baseband signals in the DUT channel by those in the loopback channel. Figure 10 shows the power and phase spectra of I/Q data after the sample division and average. Because the gain adjustment at 1.5 and 3 GHz are different amounts for DUT and RF loopback channels, the power spectrum (Figure 10a) still shows discontinuities, but the frequency response of the PLLs are canceled and become flatter. Initial phase of the TX and RX LO signals are also canceled and the phase spectrum now shows the linear change over frequency.

**Figure 11:**
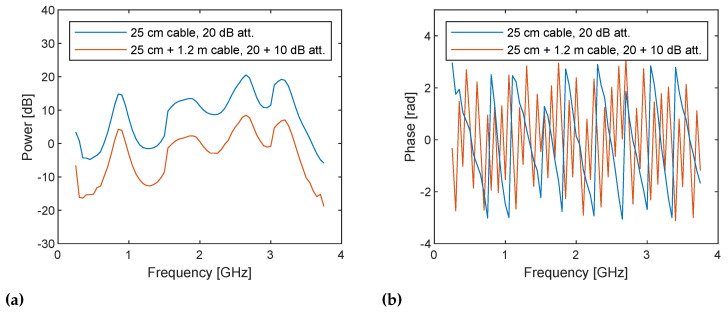
(**a**) Power spectra and (**b**) phase spectra of the uncorrected I/Q data measured at frequencies from 250 MHz to 3.75 GHz with a step size of 50 MHz for the cable through with 25 cm cable and 20 dB attenuator (blue lines) and with additional 1.2 m cable and 10 dB attenuator (red lines). The power spectra (**a**) show 10 dB down caused by the additional attenuator as expected. The phase spectra (**b**) show linear variations over frequency and the latter measurement gives faster variation as also expected due to the longer propagation path. Note that the discontinuities at 1.5 and 3 GHz in the power spectra are caused by gain adjustment.

**Figure 12:**
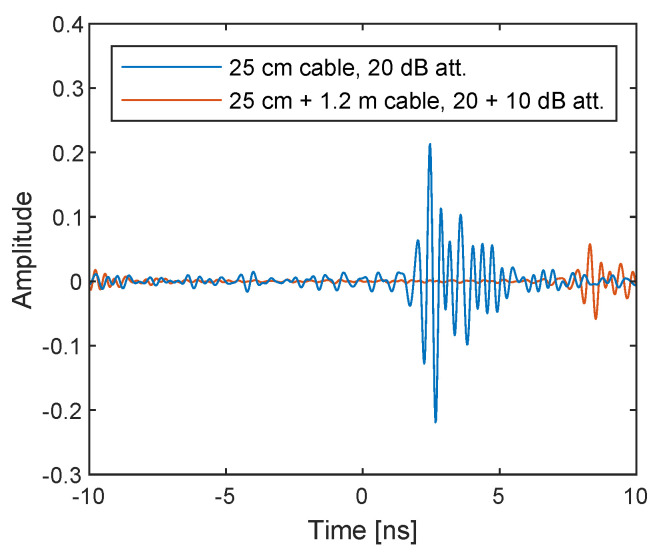
Inverse Fourier transform of the uncorrected I/Q data measured with the cable through with 25 cm cable and 20 dB attenuator (blue lines) and with additional 1.2 m cable and 10 dB attenuator (red lines). The time delay between the two pulses seem correct, but the pulses are not sharp because the spectra in Figure 11 contain the frequency response of the system.

The same postprocessing on sampled baseband signals are applied to the two measurement configurations. The obtained complex numbers correspond to the uncorrected $S_{21}$ according to Equations (11) and (12) are shown in Figure 11 as a function of the TX frequency $\omega =\omega_{\mathrm{LO}}+\omega_{b}$. It shows the system response, the difference between DUT and loopback paths to be exact, for example, due to variable gain amplifiers (VGAs) in the TX side and low noise amplifiers (LNAs) in the RX side found in Figure 1, because they are uncorrected. As expected, the 10 dB power difference due to the additional attenuator and the difference in phase cycles due to the additional 1.2 m are observed. The inverse Fourier transform of the spectra shown in Figure 12 exhibits the amplitude and the time delay difference; however, the pulses are not sharp and are noisy since uncorrected.

**Figure 13:**
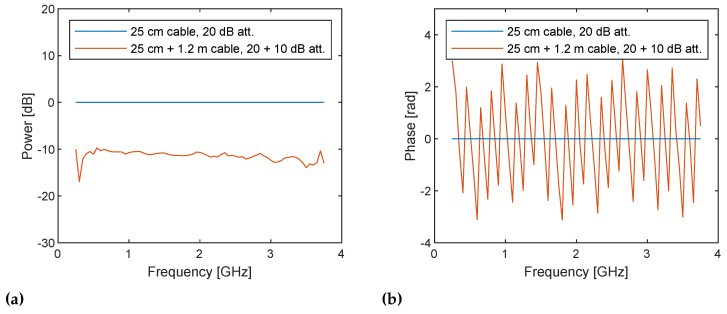
(**a**) Power and (**b**) phase spectra of the I/Q data measured at frequencies from 250 MHz to 3.75 GHz with a step size of 50 MHz for the cable through with 25 cm cable and 20 dB attenuator (blue lines) and with additional 1.2 m cable and 10 dB attenuator (red lines), corrected by the former set-up. The power spectra are flatted by correcting the system response.

To correct the system response, the complex amplitude of the second measurement (with the additional 1.2 m cable and 10 dB attenuator) is divided by that of the first measurement (with the 25 cm cable and 20 dB attenuator), which corresponds to the response calibration or through calibration for commercial VNAs, so that only the *S*-parameters of the additional DUTs in the second measurement (i.e., 1.2 m cable and 10 dB attenuator) can be obtained. The resulting power and phase spectra are shown in Figure 13. The power and phase spectra of the first measurement naturally become flat at 0 dB and 0 rad, respectively. As expected, the power spectrum of the second measurement is flattened at approximately −10 dB, which is caused by the additional 10 dB attenuator. The power in the higher frequencies is slightly decreased due to the loss in the coaxial cable. There are variations at the low- and high-frequency ends, which may be caused by unsuccessful streaming and indicates that those frequencies are probably not very accurate. The phase of the second measurement shows faster variation than that of the first measurement due to the additional 1.2 m cable.

**Figure 14:**
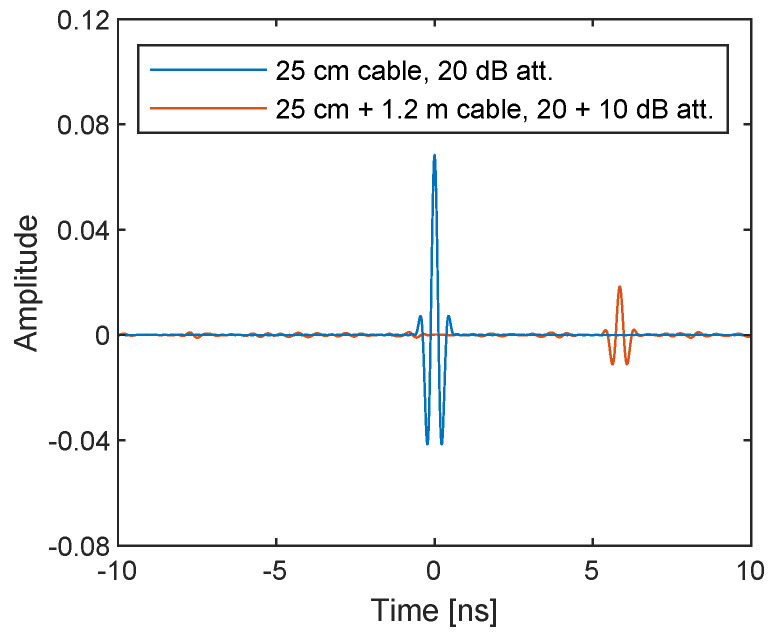
Inverse Fourier transform of the corrected I/Q data measured with the cable through with 25 cm cable and 20 dB attenuator (blue lines) and with additional 1.2 m cable and 10 dB attenuator (red lines). The pulses are sharpened compared to the uncorrected ones in Figure 12.

The corrected complex amplitude is inverse Fourier transformed to obtain the time-domain impulse response as shown in Figure 14. The result shows that two sharp pulses appeared at approximately 0 and 6 ns, which corresponds to the time for propagating through the additional 1.2 m cable. The maximum amplitudes in the first and second set-up are, respectively, 0.0684 and 0.0183, of which the ratio is 0.268, and the amplitude difference is thus −11.45 dB. It is slightly more than the amplitude drop expected by including the additional 10 dB attenuator. However, it is a reasonable value considering the attenuation in the additional 1.2 m cable, as can be seen in Figure 13a. The peak amplitudes in the first and second set-up are obtained respectively at 0 and 5.859 ns, which gives a velocity factor of 0.693. The additional 1.219 m cable uses RG-316, whose velocity factor is reportedly 0.695. Therefore, the measurement correctly reflects the time delay of a transmitted pulse, which is crucial for radar applications. From the result that the amplitude and time delay of the pulses are correctly captured, it is verified that the method to compensate for the LO phase using the internal RF loopback works and that low-cost SDRs with only one TX and RX channel can therefore be used to build UWB SFCW radar.

In the experiment, S-parameters at 71 frequencies ranging from 250 MHz to 3.75 GHz are collected to reconstruct one time-domain signal as shown in Table 2. It requires 142 TX and RX streaming in total; 71 streaming the DUT channel and 71 streaming the loopback channel because the loopback channel has to be measured every time the carrier frequency is tuned. It is the doubled number of streaming required by using high-end SDRs with more than two RX channels (e.g., one used in [22,23]). Therefore, the proposed method requires twice the time required by a more expensive solution to configure SFCW radar.

## 4. Discussion and Conclusions

The paper discussed the challenge of employing low-cost SDRs for building SFCW radar, and a solution to the challenge was presented. Low-cost SDRs typically have only one TX and RX channel, which makes it impossible to directly measure the phase of CW signals consistently. The phase is necessary for constructing SFCW radar in addition to the amplitude to reconstruct time-domain signals. The inconsistency of the phase is caused by the varying timing of the mixing of baseband and LO signals in the TX side and RF and LO signals in the RX side. The paper proposed a method to compensate for the unknown phase terms by measuring the internal RF loopback channel in addition to the DUT channel, which requires two streaming immediately in quick succession without performing frequency tuning, and dividing signals sample-wise. The operation down-converts the received baseband signals to DC, but also cancels the unknown initial phases of the TX and RX LO signals. As a result, the obtained I/Q data are referenced to the RF loopback signal, i.e., the amplitude and phase are relative to the loopback path. Because the system responses of the DUT and loopback paths are different, the obtained I/Q data need to be corrected by the cable through configuration. The experiment described in the paper shows that the proposed LO phase compensation works and that the UWB pulse can be reconstructed. Therefore, the low-cost SDR can be used to configure SFCW radar without any additional components. Because the method requires two streaming to obtain I/Q data at a frequency, it may not be suited for time-critical measurements, but still useful for prototyping a UWB radar. The method does not require any additional components; therefore, it can keep the hardware set-up simple and low-cost which makes it more accessible for prototyping and small-lot production of SFCW radar or custom-made measurement systems that provides S-parameters or time-domain response.

## Figures and Tables

**Table 1:** RF specification of a low-cost SDR, Nuand bladeRF, which is based on an RF transceiver, LimeMicro LMS6002D [27,28].

Carrier frequency range	300 MHz to 3.8 GHz
Instantaneous bandwidth	1.5, 1.75, 2.5, 2.75, 3, 3.84, 5, 5.5, 6, 7, 8.75, 10, 12, 14, 20, 28 MHz
	(programmable)
Maximum RF output power	+6 dBm (typ.)
Maximum RF input power	+23 dBm
ADC/DAC sampling rate	0.16–40 MHz
ADC/DAC resolution	12 bits
TX gain control	56 dB range, 1 dB step
TX LO leakage	−50 dBc
RX noise figure	3.5 dB (LNA1 @ 0.95 GHz), 5.5 dB (LNA2 @ 1.95 GHz),
	10 dB (LNA3 @ 1.95 GHz)
RX gain control	61 dB range, max. 1 dB step (RXVGA1), 3 dB step (RXVGA2)
Reference clock frequency	38.4 MHz
Accuracy of the reference clock	1 ppm (typ.)

**Table 2:** Set-up of the experiments.

	Single Frequency	Frequency Sweeping
LO frequency	1 GHz	249 MHz–3.749 GHz, 50 MHz step
Baseband frequency	1 MHz	1 MHz
Sampling frequency	32 MHz	32 MHz
Sampling length	4096 points = 128 μs	4096 points = 128 μs
Instantaneous bandwidth	2.5 MHz	2.5 MHz
DUT	25 cm cable, 20 dB attenuator	(1) 25 cm cable, 20 dB attenuator, (2) 25 cm + 1.219 m cable, 20 + 10 dB attenuator

## Data Availability

The data presented in this study are available on request from the corresponding author.
